# Outbreeding depression and breeding system evolution in small, remnant populations of *Primula vulgaris*: consequences for genetic rescue

**DOI:** 10.1007/s10592-017-1031-x

**Published:** 2017-12-01

**Authors:** S. Henrik Barmentlo, Patrick G. Meirmans, Sheila H. Luijten, Ludwig Triest, J. Gerard B. Oostermeijer

**Affiliations:** 1 Institute for Biodiversity and Ecosystem Dynamics, University of Amsterdam, PO Box 94062, 1090 GB Amsterdam, The Netherlands; 2 Present Address: Institute of Environmental Sciences, Leiden University, Van Steenis Building, Einsteinweg 2, 2333 CC Leiden, The Netherlands; 3 Science4Nature,, Science Park 904, 1098 XH Amsterdam, The Netherlands; 4 Laboratory for Plant Science and Nature Management, Free University Brussels, Pleinlaan 2, 1050 Brussels, Belgium

**Keywords:** Heterostyly, Homostyly, Fitness, Inbreeding depression, Heterosis, Restoration biology

## Abstract

Many species suffer from anthropogenic habitat fragmentation. The resulting small and isolated populations are more prone to extinction due to, amongst others, genetic erosion, inbreeding depression and Allee-effects. Genetic rescue can help mitigate such problems, but might result in outbreeding depression. We evaluated offspring fitness after selfing and outcrossing within and among three very small and isolated remnant populations of the heterostylous plant *Primula vulgaris*. We used greenhouse-grown offspring from these populations to test several fitness components. One population was fixed for the pin-morph, and was outcrossed with another population in the field to obtain seeds. Genetic diversity of parent and offspring populations was studied using microsatellites. Morph and population-specific heterosis, inbreeding and outbreeding depression were observed for fruit and seed set, seed weight and cumulative fitness. Highest fitness was observed in the field-outcrossed F1-population, which also showed outbreeding depression following subsequent between-population (back)crossing. Despite outbreeding depression, fitness was still relatively high. Inbreeding coefficients indicated that the offspring were more inbred than their parent populations. Offspring heterozygosity and inbreeding coefficients correlated with observed fitness. One population is evolving homostyly, showing a thrum morph with an elongated style and high autonomous fruit and seed set. This has important implications for conservation strategies such as genetic rescue, as the mating system will be altered by the introduction of homostyles.

**Electronic supplementary material:**

The online version of this article (10.1007/s10592-017-1031-x) contains supplementary material, which is available to authorized users.

## Introduction

Human activities have fragmented the habitats of many species. Habitat fragmentation increases spatial isolation and thus reduces gene flow, exposing populations to the effects of demographic, environmental and genetic stochasticity (Oostermeijer et al. 2003). Small fragmented populations are more likely to be inbred because of a higher probability of mating among close relatives, enforced selfing as a result of pollination and/or mate limitation, and increased chance of fixation through genetic drift (Young et al. 1996; Luijten et al. 2002; Edmands 2007). Inbreeding leads to higher homozygosity and increased expression of deleterious recessive alleles, which probably contributes most strongly to inbreeding depression (Charlesworth and Willis 2009). The magnitude of inbreeding depression depends on the mating system and the number of generations the population has been inbreeding (Husband and Schemske 1996; Luijten et al. 2002).

Small, isolated populations are likely to get caught in an extinction vortex of catastrophes, demographic and environmental stochasticity, genetic erosion, inbreeding depression and Allee-effects (Oostermeijer et al. 2003). Self-incompatible plant species experience additional problems, such as mate limitation due to loss of S-alleles, which may strongly reduce seed-set (Reinartz and Les 1994; Luijten et al. 2000, 2002; Pickup and Young 2008). Genetic rescue, i.e. the input of new alleles through outcrossing with other populations with whom normally no gene flow occurs, is generally considered a good strategy to increase the viability of small populations (Tallmon et al. 2004; Allendorf et al. 2013). Its effectiveness can be estimated by comparing offspring fitness components of inter- versus intrapopulation crosses. An increase in fitness can be attributed to increased heterozygosity when genetically divergent individuals are crossed. This is defined as heterosis, and usually occurs in the first generation (Tallmon et al. 2004; Allendorf et al. 2013). The opposite can also occur: a reduction in offspring performance known as outbreeding depression (Tallmon et al. 2004). This can result from mixing gene pools of ecologically and genetically distant populations, which may lead to a breakdown of epistatic (co-adapted) gene complexes associated with local adaptation (Lynch 1991; Dudash and Fenster 2000; Luijten et al. 2002; Tallmon et al. 2004; Edmands 2007; Frankham et al. 2011). As this breakdown requires recombination, it may only occur after several generations of outcrossing (Edmands 2007). There are, however, several cases of outbreeding depression observed in the F1 generation (Fischer and Matthies 1997; Etterson et al. 2007). Although increasing, experimental data on outbreeding depression are still relatively scarce compared to data on inbreeding depression.

Here, we evaluate offspring performance of three small, fragmented *Primula vulgaris* populations in the province of Drenthe, the Netherlands, as part of a genetic rescue program to save the species from regional extinction (Luijten et al. 2014). These populations are genetically strongly differentiated (*F*_*ST*_ = 0.435–0.508; van Geert et al. 2015), and separated by at least 1.5 km, which greatly reduces the probability of gene exchange (van Geert et al. 2009, 2015). Our aim was to determine (a) to what extent inbreeding and outbreeding depression and heterosis affect offspring performance, (b) how the genetic diversity of parent populations compares to their offspring, and (c) whether or not we need to worry about mixing populations during genetic rescue. To this end, we selfed and outcrossed greenhouse-grown offspring of the remnant populations and measured fitness-components of these plants and their progeny. Furthermore, we used microsatellite markers (Triest et al. 2015) to determine the degree of genetic differentiation and the inbreeding coefficients of the greenhouse-grown progeny and their parents in the remnant populations.

## Materials and methods

### Study species

Our study species was the perennial herb *P. vulgaris* (Primulaceae), which has a ‘heterostylous’ self-incompatibility system with (usually) two morphs: a long-styled ‘pin’ and short-styled ‘thrum’ (Curtis and Curtis 1985). It has recently been determined that the heterostyly in *Primula* is controlled by an S-locus supergene comprising five strongly linked genes (*CCM* ^*T*^, *GLO* ^*T*^, *CYP* ^*T*^, *PUM* ^*T*^ and *KFB* ^*T*^), which are specific for thrums and absent in pins (Li et al. 2016). Linked to the S-locus is a partial sporophytic self-incompatibility system, which further strengthens the prevention of self-fertilisation and promotes outcrossing with legitimate partners (Jacquemyn et al. 2009; Li et al. 2011). Self-compatible homostylous individuals have been observed in British populations of *P. vulgaris*, proving that both systems are susceptible to breakdown (Curtis and Curtis 1985; Boyd et al. 1990). It was previously hypothesized that homostyles arise through crossing-over (Lewis 1949; Dowrick 1956), but Li and co-workers demonstrated that they arise through a mutation (base insertion) in thrums, either in the *CYP* ^*T*^ gene—resulting in the so-called ‘long homostyle’ with a long style and high anthers—or in the *GLO* ^*T*^ gene—resulting in the ‘short homostyle’ with short style and low anthers. This also suggests that *CYP* ^*T*^ is the candidate gene for style length suppression (previously coded as *G*) and *GLO* ^*T*^ for anther elevation (the *A* gene, Li et al. 2016).

### Study populations

**Fig. 1:**
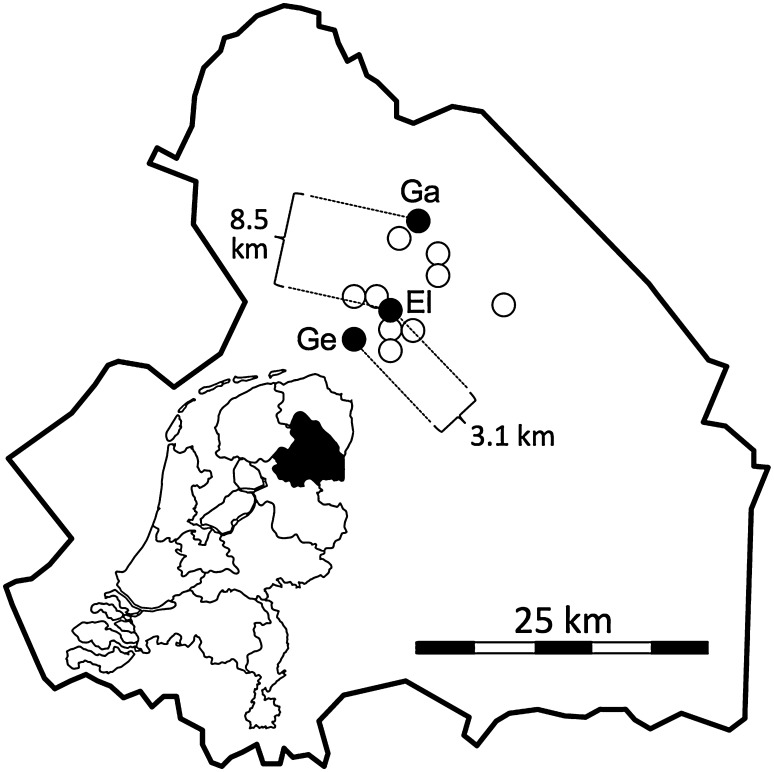
Outline of the province of Drenthe with an insert map of the Netherlands showing the location of this province in black. In the province outline, dots indicate the locations of all historically known *P. vulgaris* populations. Filled dots indicate the three extant study populations, with their abbreviated names, empty dots indicate populations that went extinct between 1950 and 2000 (n = 9). Distances between the study populations in kilometers are also shown

In the Netherlands, both the number and size of populations of *P. vulgaris* have declined strongly over the last 50–60 years, mostly as a result of land-use changes and intensification of agriculture. Currently, only three native populations are left, all situated in the province of Drenthe (Fig. 1).

**Fig. 2:**
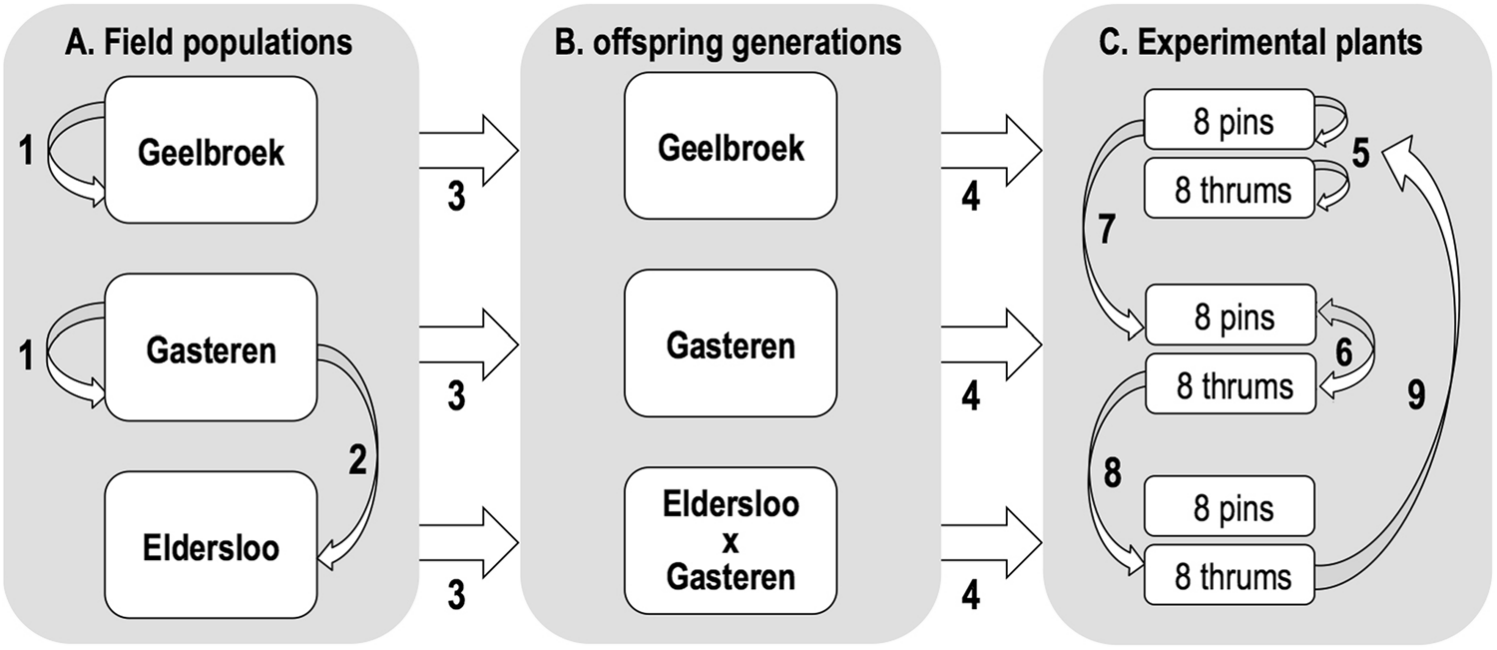
Schematic overview of the pollination experiment from the natural populations (**a**, field) to the F1 offspring (**b**, common garden) to the selected experimental plants (**c**, greenhouse). Curved arrows (1, 2, 5–9) represent pollen flow while straight arrows represent seed (3) and plant (4) transfer. Note that (1) except selfing (5), only legitimate pollinations were performed (reciprocally) and (2) pollinations 5 and 6 are repeated over all experimental plants (not shown for clarity)

The seeds producing the greenhouse-grown plants used in this study were collected from hand-pollinated flowers in the three remnant populations: Gasteren, Geelbroek and Eldersloo (Fig. 1). Owing to the distance among them, no gene flow is expected, which is corroborated by significant genetic differentiation (van Geert et al. 2015). Legitimate (pin × thrum) cross-pollinations within populations Gasteren and Geelbroek were performed manually (Fig. 2, arrows 1). As the third population Eldersloo only comprised pin individuals, these had to be outcrossed with thrums from population Gasteren to obtain seeds (Fig. 2, arrow 2); this population is hereafter referred to as El × Ga. Collected seeds were germinated in the greenhouse on wet Whatman filter paper in a Petri dish. After germination, seedlings were transferred to pots and grown in the greenhouse until they were considered strong enough to be placed outside in the common garden (Fig. 2, arrows 3).

### Experimental setup

After 1 year, when all offspring flowered, eight pin and eight thrum plants per population were randomly selected for the crossing experiment and placed randomly in the greenhouse (Fig. 2, arrows 4). The flowers that functioned as pollen recipients were emasculated by removing the anthers with fine forceps through an incision in the corolla tube, before the flower opened and before anther dehiscence. Pollen recipients were pollinated in four different treatments: (1) manual self-pollination, pollen from another flower of the same individual was brushed onto the stigma (MSP; Fig. 2, arrows 5), (2) within-family outcrossing, pollination with a random donor from the same offspring family (WF; Fig. 2, arrow 6), (3) within-population among-family outcrossing, pollination with a random pollen donor that did not share a parent with the recipient (WP; Fig. 2, arrow 6), and (4) between-population outcrossing, pollination with a random donor from another population (BP; Fig. 2, arrows 7, 8 and 9). For the latter treatment, pollen recipients from population Gasteren were outcrossed with population Geelbroek (Fig. 2, arrow 7), pollen recipients from population El × Ga were backcrossed with population Gasteren (Fig. 2, arrow 8) and pollen recipients from population Geelbroek were outcrossed with population El × Ga (Fig. 2, arrow 9). Each experimental plant received all four pollination treatments and each treatment was replicated four times, resulting in an effective sample size of n = 8 × 4 = 32 per treatment and a total of 3 populations × 2 morphs × 8 plants × 4 treatment types × 4 replicates = 768 pollinations. In the rare case that a plant did not produce enough flowers to allow all treatments, an additional plant from the same family was used. In total, 754 out of the planned 768 pollinations were successful (i.e. pollen was transferred successfully and pollinated flowers didn’t wither). After pollination, fruit set was assessed. For each ripe fruit, the numbers of viable and aborted seeds (small and shrivelled, but with a dark brown seed coat) were counted. Mean seed weight per fruit was calculated as the weight of all viable seeds together divided by the number of viable seeds. Furthermore, for each morph and population, the number of unfertilised seeds (visible as tiny, shrivelled ovules) was counted for 20 fruits. The total population seed set per morph was calculated by using the sum of the numbers of viable, aborted and unfertilised seeds as an estimate of the initial number of ovules per flower. Cumulative fitness (‘seed yield’) was calculated by multiplying fruit set x seed set x mean seed weight.

### DNA extraction and microsatellite analysis

In 2008, plants were sampled from the remnant populations Geelbroek (n = 11), Gasteren (n = 21) and Eldersloo (n = 3) respectively (van Geert et al. 2015). Six years later, an additional ten adult plants and three juveniles were sampled from the populations Gasteren and Geelbroek respectively. Because we knew the identity of each individual, we could avoid resampling the same individuals. In addition, 30 individuals were sampled from the offspring of each of the native populations. This resulted in a total of 138 samples across the three natural and three greenhouse-grown populations. DNA was extracted from young leaf material, that was either dried or frozen with liquid nitrogen, using an adjustment of the CTAB method described by Doyle and Doyle (1987). In addition, 20 µl of 3M sodium acetate was added, after the RNAse incubation, to all samples before rinsing them with EtOH and drying them in a vacuum exsiccator. After DNA extraction, microsatellite analysis was performed as described in Triest et al. (2015). GeneMarker version 2.2.0 (SoftGenetics LLC.) was used to score the alleles at each primer. Thirteen of the 14 microsatellite loci gave interpretable results. One monomorphic locus was excluded, resulting in a total of 12 loci used for analysis.

### Statistical analysis

Results of the pollination experiments were tested with linear mixed-effect models (ANOVA and ANCOVA) using R (v3.1.1; R Core Team 2014). The factors used in the analyses were population (3 levels), morph (2 levels) and treatment (4–5 levels). As multiple replicates per treatment per maternal plant were performed, a nested factor was incorporated in the model to account for the effect of plant individual. Type-III errors were applied to all models to generalize the effects of population, morph and treatment and their respective interaction effects. For all models, backwards selection was performed using Akaike’s Information Criterion (AIC) or insignificant effects of the variables. Normality of the model variables and residuals was evaluated with QQ-plots. An *lme* model with seed weight as response variable and number of seeds produced per plant as explanatory variable showed that an increasing number of seeds produced per plant significantly negatively affected the mean seed weight. Therefore, we corrected for this maternal effect by using the residuals of this model in further (statistical) analysis on seed weight, such as the calculation of the cumulative fitness. Cumulative fitness was tested with an *lme* model. Model residuals and random variables were normally distributed. The assumption of homogeneity of variances was violated at the level of population and treatment (Levene’s test, p < 0.05), however, transformation (log or square root) of the data did not improve this.

Allelic richness, expected and observed heterozygosity, *F*_*IS*_ and *F*_*ST*_ (AMOVA, 999 permutations) were calculated with GenAlEx v6.501 (Peakall and Smouse 2006, 2012). There was no difference in these parameters among the (few) plants sampled from population Geelbroek in 2008 and 2014 and therefore these samples were pooled. Samples from 2008 to 2014 from Gasteren were significantly different, however, and were therefore analyzed separately. Allelic richness and expected heterozygosity were compared between populations/generations using the results per locus as replicates. Wilcoxon matched pairs tests were used, as the data for both parameters were not normally distributed.

## Results

### Pollination experiment

**Fig. 3:**
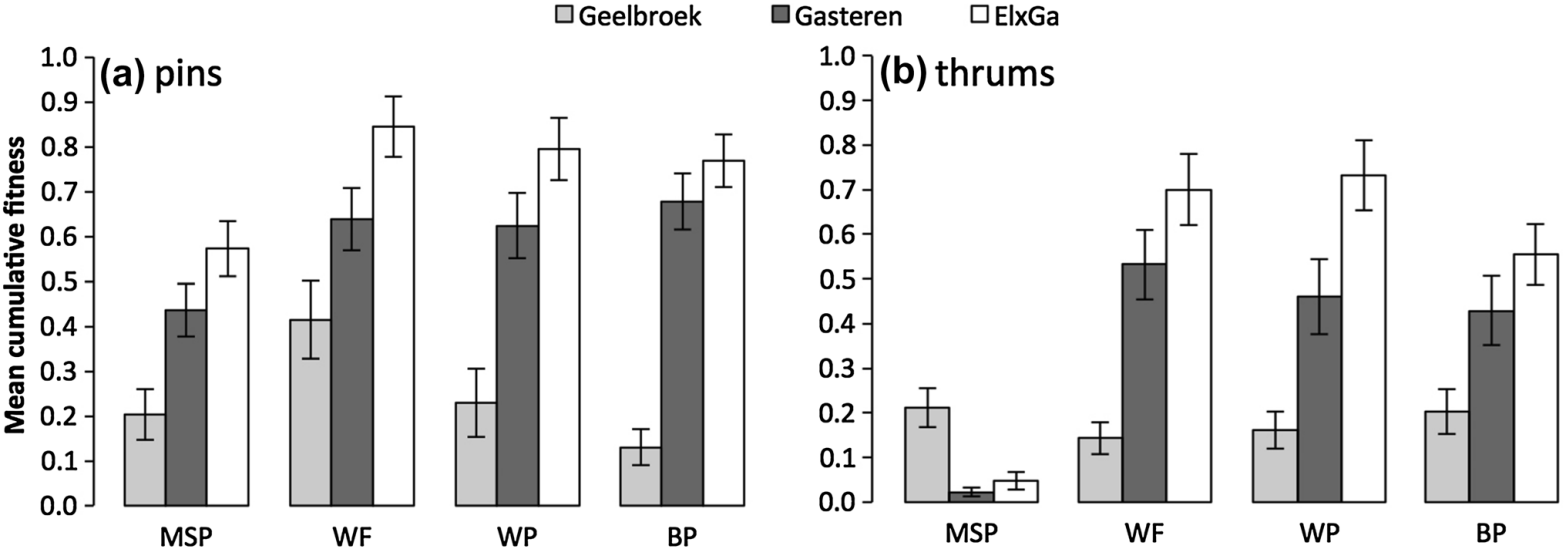
Mean cumulative fitness (fruit set × seeds/seed set × mean seed weight) for **a** pins (n = 27–32) and **b** thrums (n = 30–32). Error bars represent the standard error. *MSP* manual self-pollination, *WF* within-family, *WP* within-population, *BP* between-population. Note that the autonomous self-pollination ‘treatment’ is not included, as there was no data on its fruit set

Both population and treatment had a significant effect on cumulative fitness (df = 2, *p* < 0.001; df = 3, *p* < 0.001 respectively). Within each treatment, except for manual selfing of thrums, cumulative fitness was highest in population El × Ga (Fig. 2a, b). This difference was mostly caused by seed weight (Supporting information, Fig. S1–S4), which was highest in all El × Ga treatments. Between-population crosses showed the lowest cumulative fitness in Geelbroek pins compared to the other pin crosses (Fig. 3a). This resulted from consistent differences in fruit set, seed set and seed weight (Supporting information; Figs. S1, S2, S4 respectively). Between-population crosses also yielded lower cumulative fitness in El × Ga thrums compared to within- and between-family crosses within that population (Fig. 3b). This difference was mainly caused by lower seed set and seed weight (Supporting information; Fig. S2 and S4 respectively). Mean cumulative fitness was higher for manual selfings of pins than for thrums from populations Gasteren and El × Ga, but equal for population Geelbroek (Fig. 3a, b). Mean cumulative fitness was higher in nearly all treatments of pins compared to thrums (Fig. 3a, b), even though the factor morph was not quite significant (*p* = 0.075). Significant interactions were found for population × treatment, morph × treatment and population × morph × treatment (*p* < 0.05, *p* < 0.001 and *p* < 0.001 respectively).

High fruit set was sometimes observed on flowers that were not emasculated and received no pollination treatment. We considered these produced by autonomous self-pollination (ASP). In Gasteren and El × Ga pins, autonomously self-pollinated fruits were observed for 27 and 50% of the maternal plants, respectively. The mean number of autonomously self-pollinated fruits per Gasteren and El × Ga pin individual was 0.54 (SE 1.21) and 0.50 (SE 0.74), respectively, whereas no autonomously self-pollinated Geelbroek pins produced a fruit. By contrast, 100% of the Geelbroek thrums produced autonomously self-pollinated fruits with a mean number of 5.08 (SE 3.20) fruits per plant, against 0.00 (SE 0.00; 0 fruits in total) and 0.11 (SE 0.33; 1 fruit in total) for Gasteren and El × Ga thrums, respectively. As these fruits were unexpectedly observed during the experiment, we could not collect data on the proportion of ASP fruit set as we didn’t count the total no. of flowers/plant. Therefore, cumulative fitness of the ASP “treatment” could not be calculated. Fruit set (in numbers/plant) of the ASP treatment was significantly higher for Geelbroek thrums compared to all other populations and morphs (ANOVA with Tukey post-hoc test: *p* < 0.001 for all comparisons) with an expected significant interaction between population and morph (*p* < 0.001).

### Genetic analysis

#### Within-population genetic variation

Allelic richness (*A*) of the natural populations was low (between 1.25 and 1.67) and comparable between populations. No differences in *H*_O_ and *H*_E_ were observed. A negative inbreeding coefficient was observed for the natural population Eldersloo (*F*_*IS*_ = − 0.88, Table 1). However, this value was based on only three plants and three loci as the population was monomorphic for the other nine loci. By contrast, *F*_*IS*_ was close to zero for the natural population Gasteren (− 0.05) and slightly negative for Geelbroek (− 0.19, Table 1).

**Table 1:** Within-population genetic variation of the remnant natural populations (field) and the manually outcrossed F1 generations of these populations

Population	N	Pin frequency	*A*	*H*_*O*_	*H*_*E*_	*F*_*IS*_
Gasteren field SE	21	0.71	1.67	0.26	0.25	− 0.05
			0.19	0.07	0.07	0.08
Gasteren F1 SE	30	0.59	1.75	0.21	0.24	0.12
			0.22	0.07	0.07	0.13
Eldersloo field SE	3	1.00	1.33	0.31	0.16	− 0.88
			0.14	0.13	0.07	0.07
El × Ga F1 SE	30	0.55	2.75	0.64	0.44	− 0.40
			0.33	0.09	0.06	0.10
Geelbroek field SE	14	0.71	1.25	0.08	0.07	− 0.19
			0.13	0.04	0.04	0.01
Geelbroek F1 SE	30	0.50	1.830.27	0.080.04	0.11	0.46
					0.04	0.15

All reported values are means with their respective standard errors (SE)

Allelic richness was larger for all offspring compared to their parent populations, with the largest difference between El × Ga and Eldersloo. *H*_*E*_ of the El × Ga F1 (0.44, Table 1) was higher than that of the natural populations Eldersloo (0.16, *p* < 0.01) and Gasteren (0.25, *p* = 0.07), although the latter comparison was not significant (Table 1). The same was found for *H*_*O*_, but only marginally significant (Eldersloo/Gasteren x Eldersloo vs. Gasteren, *p* = 0.06/*p* = 0.05 respectively). In comparison, *H*_*E*_ also increased significantly (*p* = 0.02) from 0.07 for the natural population Geelbroek to 0.11 for its F1 offspring population in the greenhouse. Such an increase was not observed for *H*_*O*_. No deviations in either *H*_*E*_ or *H*_*O*_ were observed for the population Gasteren offspring compared to their parent population.

While the inbreeding coefficient of the F1 offspring El × Ga increased to − 0.40 relative to its parent population Eldersloo (− 0.88), it decreased relative to the other parent population, Gasteren (− 0.05, Table 1). For population Geelbroek, *F*_*IS*_ increased from − 0.19 in the parents to 0.46 in the offspring (Table 1). *F*_*IS*_ also increased from − 0.05 for the natural population Gasteren to 0.12 and 0.14 (Supporting information, Table S6) for the greenhouse and field offspring respectively. These changes could however not be confirmed statistically due to the large standard errors.

### Between-population genetic differentiation

High *F*_*ST*_ values were observed among the natural populations, as well as among their offspring generations in the greenhouse. However, *F*_*ST*_ values between Gasteren and its offspring and Geelbroek and its offspring were close to zero (Table 2). As expected, between-population genetic differentiation of the offspring was low between Gasteren and the F1-population El × Ga and between Eldersloo and El × Ga (Table 2). The differentiation between the offspring generations of the natural populations Gasteren and Geelbroek (0.674) was similar to that between their parent populations (0.698, Table 2). However, *F*_*ST*_ decreased between the offspring populations of Geelbroek and El × Ga (0.556) compared to the parent populations Geelbroek and Eldersloo in the field (0.869, Table 2). Furthermore, *F*_*ST*_ also decreased between the offspring populations from El × Ga and Gasteren (0.295) compared to the differentiation between the natural populations Eldersloo and Gasteren (0.661, Table 2).

**Table 2:** Between-population genetic differentiation (*F*_*ST*_) and respective *p*-values (in italics) of the remnant natural populations (field) and manually outcrossed F1 generations of these populations

	Gasteren Field	Gasteren F1	Geelbroek Field	Geelbroek F1	Eldersloo Field	El × Ga F1
Gasteren field		*0.011*	*0.001*	*0.001*	*0.001*	*0.001*
Gasteren F1	0.031		*0.001*	*0.001*	*0.001*	*0.001*
Geelbroek field	0.698	0.715		*0.382*	*0.001*	*0.001*
Geelbroek F1	0.658	0.674	− 0.003		*0.001*	*0.001*
Eldersloo field	0.661	0.673	0.869	0.750		*0.002*
El × Ga F1	0.276	0.295	0.572	0.556	0.233	

### Fitness related to heterozygosity and inbreeding

**Fig. 4:**
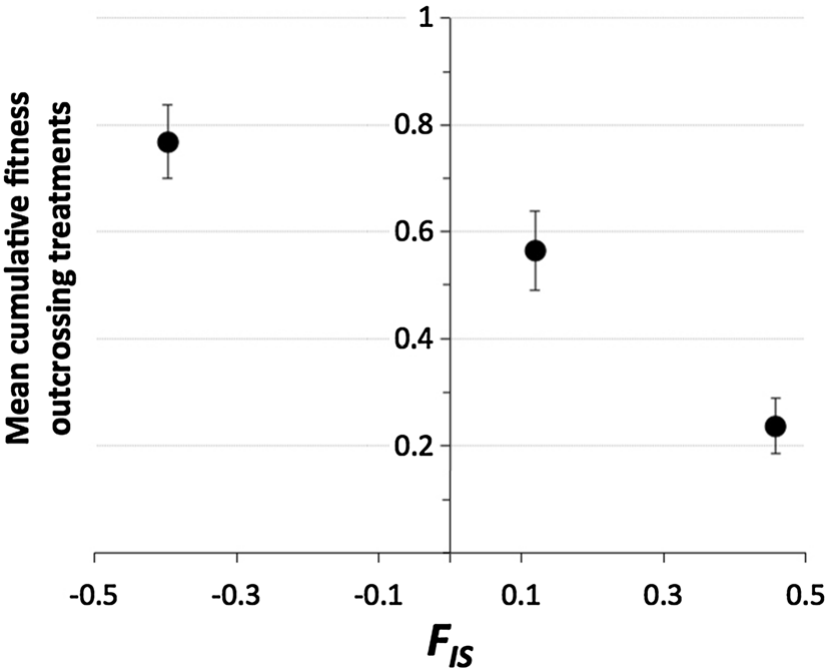
Mean cumulative fitness calculated over all within-population outcrossing treatments in the three offspring populations relative to the inbreeding coefficient, *F*_*IS*_, determined from 12 microsatellite loci

Overall, cumulative fitness was lowest in Geelbroek, followed by Gasteren, and highest in El × Ga with *H*_*O*_-values of 0.08, 0.25 and 0.64 for their offspring populations respectively. This indicates a positive relationship between cumulative fitness and *H*_*O*_. A reverse pattern was observed for cumulative fitness related to *F*_*IS*_ with values of 0.24, 0.12 and − 0.44 for Geelbroek, Gasteren and El × Ga, respectively (Fig. 4).

## Discussion

### Inbreeding and outbreeding depression

We studied the fitness of in- and outbred progeny of three small, remnant *P. vulgaris* populations. Currently, there is probably no gene flow among these populations and they are genetically strongly differentiated (van Geert et al. 2015; this study). Therefore, we hypothesized that outcrossing among the offspring of these remnant populations might result in outbreeding depression. Indeed, significant outbreeding depression was found for El × Ga thrums, as the fitness after between-population outcrossing was lower than the within-population outcrosses. This drop in cumulative fitness could not be confirmed for Gasteren thrums, which are closely related to El × Ga thrums. Outbreeding depression is often more likely to occur in later generations when epistatic (coadapted) gene complexes start to break up due to recombination (Edmands 2007). Therefore, it is possible that the offspring population El × Ga exhibited outbreeding depression, because in contrast to the other treatments it concerned the second instead of first generation of interpopulation outcrossing. It is less likely that this reduced fitness can be attributed to a decline in heterosis (Tallmon et al. 2004; Allendorf et al. 2013), in which case the cumulative fitness would also have been lower after within-population crosses.

Reductions in fitness due to outbreeding after (sub)population outcrossing have been observed in other species. Comparable results were obtained in *Lotus scoparius* (Montalvo and Ellstrand 2001) and *Gymnadenia conopsea* (Sletvold et al. 2012). In contrast to these studies we also compared seed set to a within-family cross. For Geelbroek pins, within-family crosses yielded higher seed set, seed weight and cumulative fitness than between-family and between-population crosses. To a lesser extent, the fitness of between-population crosses was also lower than within-population crosses in the El × Ga F1 plants, although here within- and between-family fitness was similar. Hence, there was an inverse relationship between relatedness of pollen donor and recipient and resulting offspring fitness. These findings suggest that some outbreeding depression already occurred in a very early stage of the life cycle. In particular in the second-generation offspring of the El × Ga population, this may have been caused by the breakdown of co-adapted (or perhaps rather “co-drifted”) gene complexes after a first round of recombination (Edmands 2007; Frankham et al. 2011). We did not expect such differences in adaptation among these three populations, as they have only been fragmented and isolated for 3–5 decades. Population Geelbroek flowered several weeks later than populations Gasteren and Eldersloo, in the field as well as in the greenhouse, suggesting a genetic basis for this difference. An alternative explanation for the observed low fitness of between-population crosses in Geelbroek could be that the pollen of Gasteren × Eldersloo donor flowers was relatively old while the stigmas of Geelbroek flowers were still relatively young. However, this cannot explain the lower fitness of among—relative to within-family crosses within population Geelbroek.

### Heterosis

Regardless of the occurrence of outbreeding depression, cumulative fitness was consistently highest in El × Ga compared to the other populations for nearly all treatments. This offspring generation was obtained by crossing two genetically differentiated populations in the field. Hence, increased heterozygosity in the progeny is very likely and *H*_O_ was indeed highest. We therefore attribute the observed higher fitness to heterosis (Edmands 2007). This also indicates that the remnant populations Eldersloo and Gasteren were inbred. Heterosis was mostly expressed as increased fruit set and seed weight, as seed set was comparable to the closely related offspring population Gasteren. In pins, fruit set after the different treatments per population was similar. However, in El × Ga thrums, fruit set was higher than in the—closely related—Gasteren thrums in all treatments, indicating heterosis. Furthermore, although seed weight decreased with decreasing relatedness of the parents in Geelbroek pins, Geelbroek thrums showed a higher seed weight after interpopulation crossing. This can also be interpreted as heterosis (Edmands 2007).

### Morph-specific responses

Morph-specific responses were found for all tested fitness components, and were also found by van Geert on the same species (2010). Furthermore, van Rossum et al. (2006) showed that in small populations of *Primula veris*, seed set of pins was higher than for thrums, whereas in large populations it was the reverse. They gave two hypotheses for this: (1) higher accessibility of pin stigmas and thrum anthers to pollinators and (2) morph-specific differences in the partial SI system, i.e. a higher degree of self-compatibility within pins. While these hypotheses may hold in an open-pollination study, the first hypothesis does not apply to our study, because we used hand pollinations so that accessibility to pollinators played no role. The second hypothesis might explain differences between the selfing treatments, but morph-specific responses were also found in legitimate crosses where emasculation ruled out selfing. A possible explanation for morph-specific responses might be linkage between genes affecting fitness components. Such linkage is likely on the chromosome holding the *S-*locus (Li et al. 2016), and would explain morph-specific differences in fruit and seed set.

### Homostyly

We show that the SI system reported for *P. vulgaris* (Jacquemyn et al. 2009) is not infallible. Depending on population and style morph, manual and autonomous selfing showed substantial seed set. In the greenhouse, no pollen vectors such as thrips were observed, so pollinations occurred either manually or autonomously. The highest fruit set after manual selfing (56%) was found in Geelbroek thrums, compared to 16 and 28% for Gasteren and El × Ga thrums, respectively. Fruit set of manually self-pollinated Geelbroek thrums was also higher than all other pollination treatments on either pins or thrums in Geelbroek. Furthermore, most Geelbroek thrums in the field and all greenhouse-grown ones showed elongated styles. These appeared to be about as long as typical pin styles and stigmas were often observed pressing against the anthers right after or even before anthesis. This most likely resulted in the significantly higher number (on average 5 per plant) of autonomously self-pollinated fruits on Geelbroek thrums compared to the other populations. Seed set after autonomous and manual selfing in thrums was also higher in Geelbroek than in the other two populations.

Combining these findings, it is likely that both the heteromorphic and sporophytic self-incompatibility system are partially broken down in the Geelbroek thrums. Hence, the latter have most likely evolved into so-called ‘long homostyles’ (Gilmartin 2015). Homostyles have been reported from the United Kingdom in “large densities” by Curtis and Curtis (1985), and by Piper et al. (1984) and Boyd et al. (1990) who showed that they are self-fertile. Furthermore, homostyles can fertilize heterostyles (Boyd et al. 1990; Richards 1997), which is corroborated by our study. The long homostyle phenotype is caused by a mutation (base insertion) in the *CYP* ^*T*^ (previously coded as G) gene within the *S*-locus that controls style length (Li et al. 2016). The presence of this mutant genotype in Geelbroek would explain the high rate of autonomous selfing. Porcher and Lande (2005) showed theoretically that breakdown of the self-incompatibility system can be advantageous under a wide range of conditions, one of which is pollen limitation. As the latter often occurs in small and isolated populations (Oostermeijer et al. 2003), it is likely that the evolution towards homostyly observed in this study was facilitated by selection under conditions of severe pollination limitation (Busch and Schoen 2008).

### Genetic diversity and inbreeding coefficients

Because we manually outcrossed plants from the remnant populations we expected that genetic diversity would increase or be maintained. Indeed, allelic richness either increased or remained equal in the offspring populations compared to their parent populations in the field. Unexpectedly, *F*_*IS*_ values of the Gasteren and Geelbroek offspring populations increased relative to their parent populations (Table 1). *F*_*IS*_ in these populations actually became positive instead of approaching zero, indicating inbreeding. Possibly, this can be explained by biparental inbreeding, because the remnant populations were very small but had a high density. Because of genetic neighbourhood structure in the species (Jacquemyn et al. 2009), it is quite likely that the outcrossed individuals are closely related, explaining increasing inbreeding coefficients in the outcrossed offspring populations.

All three remnant populations showed a heterozygote excess (Table 1). This may simply result from the fact that self-incompatibility increases the likelihood of mating between individuals carrying different alleles (Stoeckel et al. 2006). In addition, it has been hypothesized that when large populations of perennial plants decrease in size, heterozygotes may show the highest survival due to higher masking of deleterious recessive alleles (i.e. exhibiting the lowest inbreeding depression). As a result, a regressive population of surviving adults (Oostermeijer et al. 1994), would exhibit an excess of heterozygotes (Tonsor et al. 1993; Oostermeijer et al. 1995, 2003; Stoeckel et al. 2006). Van Geert et al. (2008) found that naturally established offspring in populations of *P. vulgaris* were more inbred than their parents. This is in line with the increasing *F*_*IS*_ we observed, although observed heterozygosity of our offspring populations Geelbroek and Gasteren was very similar to their respective parent population. The increase in heterozygosity of the offspring population El × Ga is best attributed to outcrossing populations Gasteren and Eldersloo, and is in line with the observed heterosis. Overall, the results showed that cumulative fitness was positively associated with higher heterozygosity and negatively with the inbreeding coefficient. Similar positive relationships between heterozygosity and fitness were observed in offspring of *Gentiana pneumonanthe* (Oostermeijer et al. 1995) and *Arnica montana* (Oostermeijer et al. 2003). Jacquemyn et al. (2003) did not find any significant heterozygosity-fitness correlations in Belgian populations of *P. vulgaris*, probably because they determined fitness parameters (size, no. of flowers) on adult plants in the field, for which environmental effects could not be excluded.

### Implications for conservation

As outbreeding depression in small and isolated populations of *P. vulgaris* might counteract the positive effects of genetic rescue, it is important to view the current findings in the light of this conservation strategy. While heterosis occurred in the offspring population El × Ga, the backcrosses to Gasteren yielded statistically significant outbreeding depression. However, overall cumulative fitness of the offspring population El × Ga was still highest, indicating that fitness still increased as a result of genetic rescue. This suggests that artificial inter-population crossing and reintroduction of outbred seed mixtures is a good strategy for conservation of *P. vulgaris*. Of course this study only considers the early stages of the life cycle. A follow-up field experiment is being carried out to study subsequent fitness-components, reflected in the demographic vital rates: survival, growth and reproduction.

It is extremely interesting that one of the three small, isolated populations is in the process of evolving homostyly, showing breakdown of self-incompatibility and heterostyly. One clear benefit is that pollinators are no longer required for seed production, as homostyles are highly capable of autonomous selfing (this study, Piper et al. 1984). The most important—long-tongued—pollinators of *P. vulgaris*—*Bombus hortorum* and *Anthophora plumipes*—were not observed in any of the three remnant populations, which implies that the populations might benefit from being able to produce seeds through autonomous selfing to prevent extinction. A clear drawback of only producing seeds through selfing is increased inbreeding resulting in inbreeding depression (Boyd et al. 1990). This could lower the fitness of homostyles in following generations and compromise population viability due to inabilities to adapt to, e.g. changes in habitats and climate. Even though the genetic load is expected to be high in a normally obligatory outcrosser (Charlesworth and Willis 2009), the existence of thriving homostyle populations in the UK (Curtis and Curtis 1985) implies that it is possible to overcome the initial inbreeding depression. Most likely, this is strongly linked to habitat suitability and population growth rates: in rapidly growing populations in good habitat, there will be large numbers of seeds germinating and establishing. Under these conditions, strong selection against inbred seedlings does not lower overall recruitment rates too much. Selection might then be more effective in purging the genetic load, so that further inbreeding is less detrimental. Knowledge of this selection process is of key importance for genetic rescue projects involving self-incompatible and/or heteromorphic plants. In our current genetic rescue program for *P. vulgaris*, it needs to be decided whether or not to include homostyles in the source material for the reintroduction of new populations. We argue that we should include hetero- as well as homostyles and let natural selection determine which geno- and phenotypes have the highest fitness. The outcome will probably depend on the local availability of suitable pollinators, habitat quality and the strength of inbreeding and outbreeding depression. As a follow-up to this study, the evolutionary-ecological dynamics of ten offspring populations, introduced recently at different sites in the same nature reserve as the parent populations, will be monitored in detail over the coming years, and will undoubtedly provide many novel insights into inbreeding and outbreeding effects later in the life cycle (e.g. growth and flowering).

## Electronic supplementary material

Below is the link to the electronic supplementary material.

Supplementary material 1 (DOCX 610 KB)
