# Supplementary material for: Outbreeding depression and breeding system evolution in small, remnant populations of Primula vulgaris: consequences for genetic rescue
Source: Conserv Genet. 2017 Dec 1;19(3):545–54. doi: 10.1007/s10592-017-1031-x (PMC6448329; doi:10.1007/s10592-017-1031-x)
Supplement: Supplementary file 1 — Supplementary material 1 (DOCX 610 KB) [file 10592_2017_1031_MOESM1_ESM.docx]

**Supporting information Barmentlo et al. COGE-D-16-00288**

***Statistical analysis***

To test for differences in fruit set, a generalized linear multivariate model using Penalized Quasi-Likelihood was used (glmmPQL, R-package ‘MASS’). The *glmmPQL* function allows for mixed-effect modelling of logistic data by approximating the data and giving a penalty to the random effect. The variables seed set and number of aborted seeds were divided by their respective average seed set per morph and population to correct for morph and population-specific responses. The resulting fractions were analysed by linear mixed-effect models (lme, R-package ‘*nlme*’). The variables fruit set and seeds/seedset met the required normality assumptions. After squareroot transformation of the data, these assumptions were also met for the variable aborted/seedset. Homogeneity of variances, tested by Levene’s test for homogeneity (*p*>0.05), was observed for all three variables.

A Pearson’s test was performed to test for correlation between the number of seeds per fruit and the average seed weight. The residuals of a regression between the number of seeds (nested by maternal plant) and seed weight were then added to the *lme* model for seed weight to account for the maternal effect on seed weight. After logarithmic transformation of the variables average seed weight and number of seeds per fruit, the residuals of the model for average seed weight, as well as the random variable, shifted towards a normal distribution. Homogeneity of variances was found at the level of population and treatment (Levene’s test, *p*>0.05) for this model, but not at the level of morph (Levene’s test, *p*<0.05).


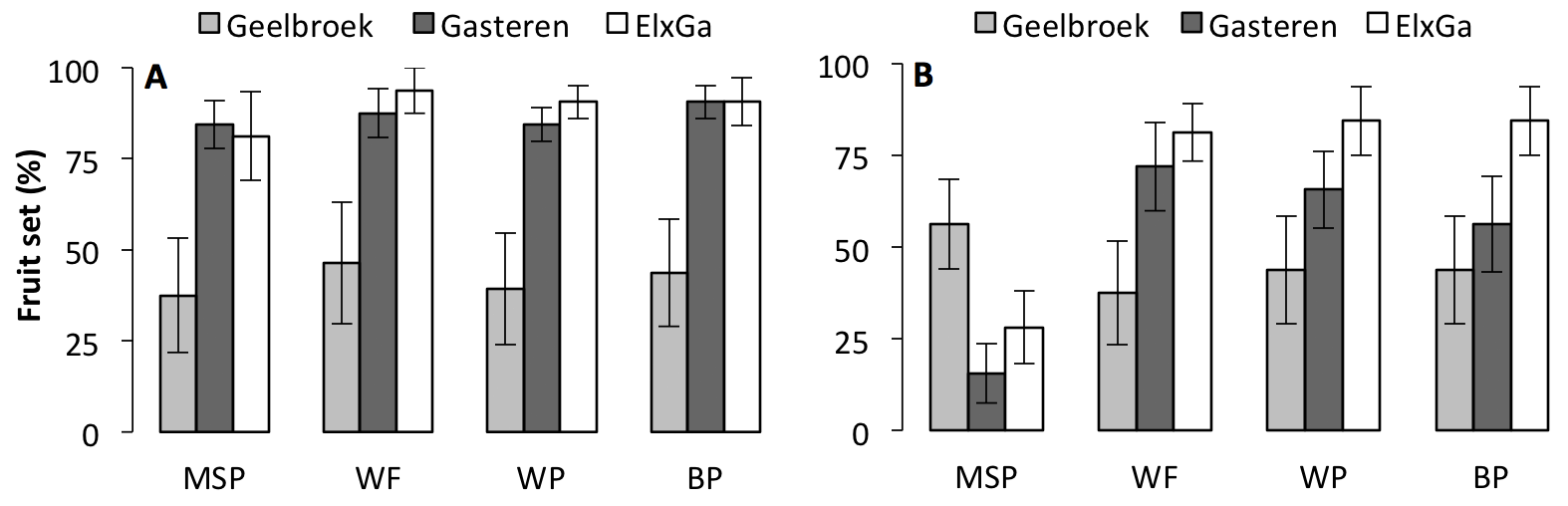


**Figure S1.** Mean fruit set (% of total performed selfings and outcrosses) per treatment for the pin (A) and thrum (B) morph. Data is shown as the average of the plant means per treatment (n = 8). Error bars represent the standard error. MSP: ‘Manual Self-Pollination’, WF: ‘Within-Family’, WP: ‘Within-Population’, BP: ‘Between-Population’.

**
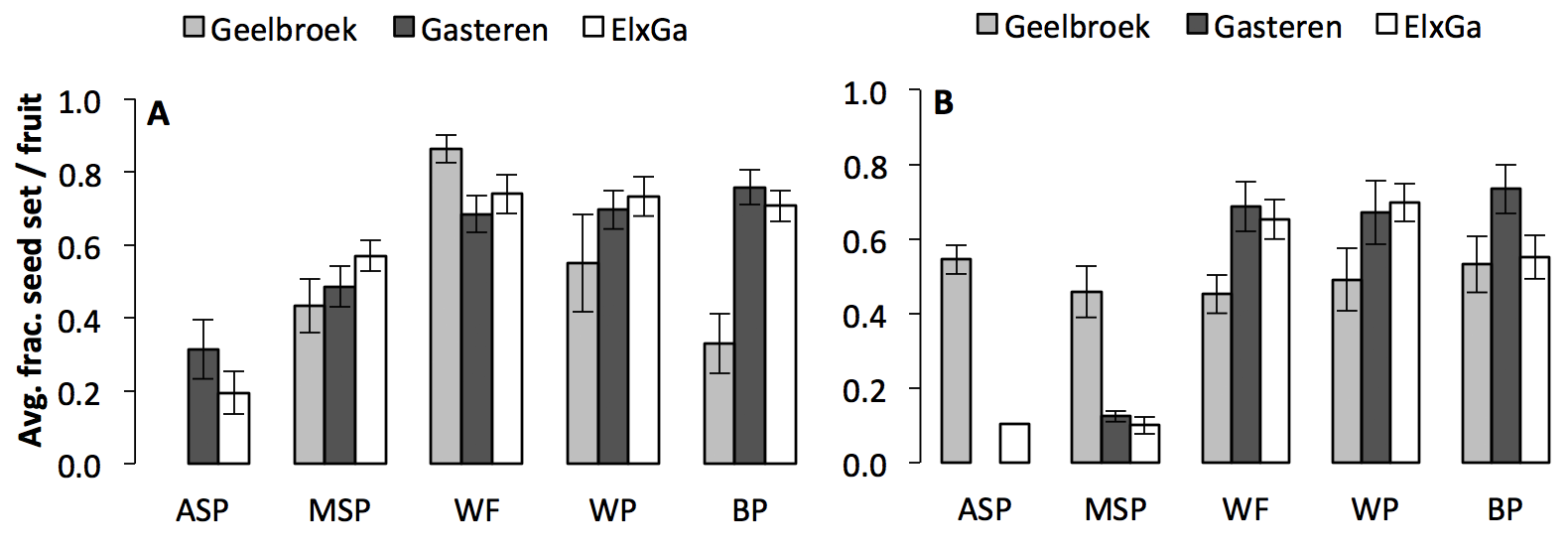
Figure S2.** The mean seed set (fraction of seed set divided by the average possible seed set) per fruit for pins (A, n = 4 – 30) and thrums (B, n = 1 – 65). Error bars represent the standard error. ASP: ‘Autonomous Self-Pollination’, MSP: ‘Manual Self-Pollination’, WF: ‘Within-Family’, WP: ‘Within-Population’, BP: ‘Between-Population’.

**
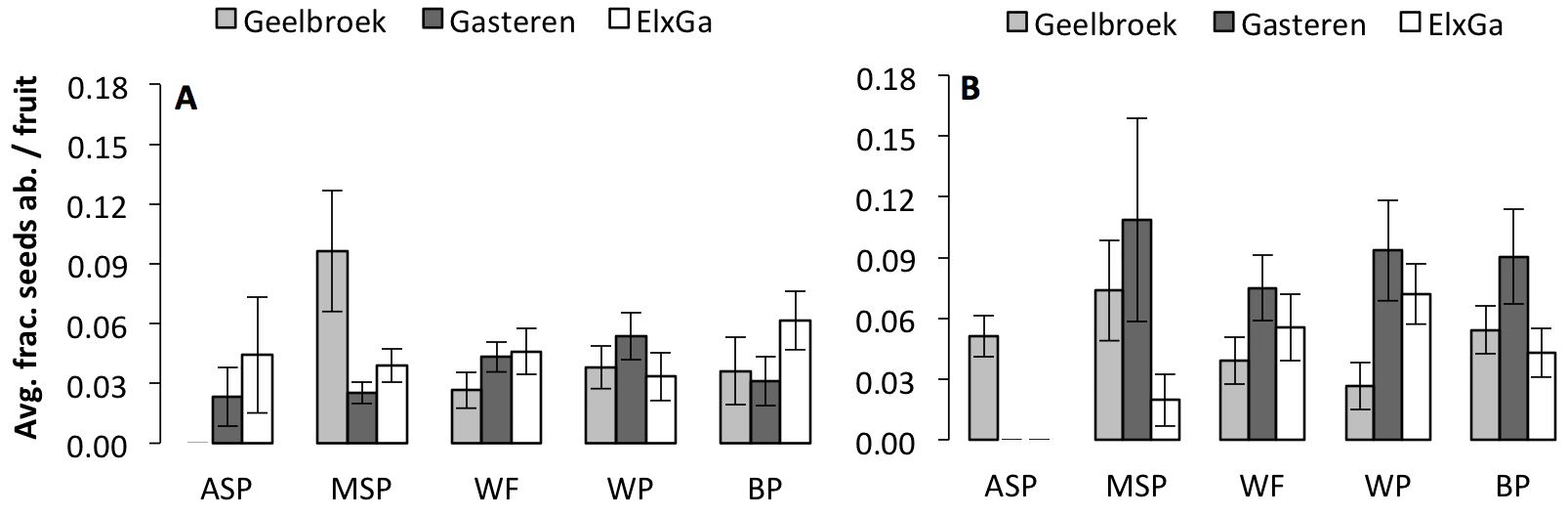
**

**Figure S3.** The mean fraction seeds aborted (divided by the average possible seed set) for pins (A, n = 4 – 30) and thrums (B, n = 1 – 65). Error bars represent the standard error. ASP: ‘Autonomous Self-Pollination’, MSP: ‘Manual Self-Pollination’, WF: ‘Within-Family’, WP: ‘Within-Population’, BP: ‘Between-Population’.

**
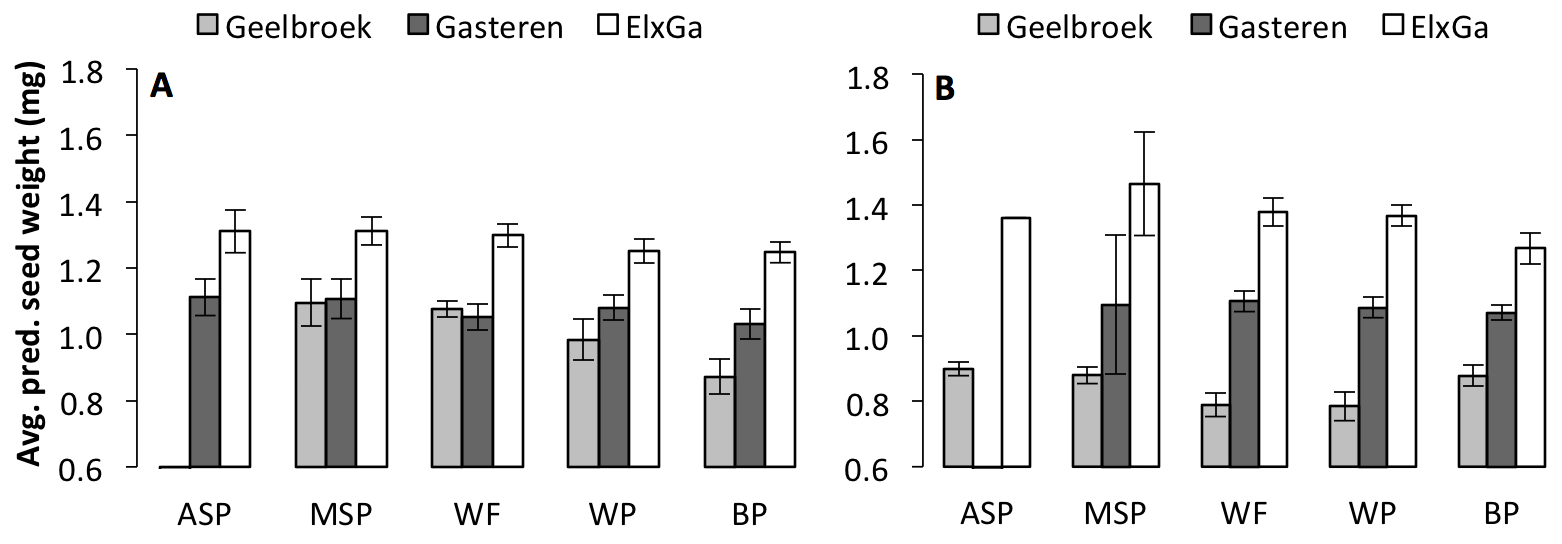
**

**Figure S4.** The mean predicted seed weight (mg) for pins (A, n = 4 – 30) and thrums (B, n = 1 – 65). Error bars represent the standard error. ASP: ‘Autonomous Self-Pollination’, MSP: ‘Manual Self-Pollination’, WF: ‘Within-Family’, WP: ‘Within-Population’, BP: ‘Between-Population’. Predicted rather than actual values are shown to exclude the effect of the number seeds on the average seed weight. For clarity, untransformed predicted means are shown.


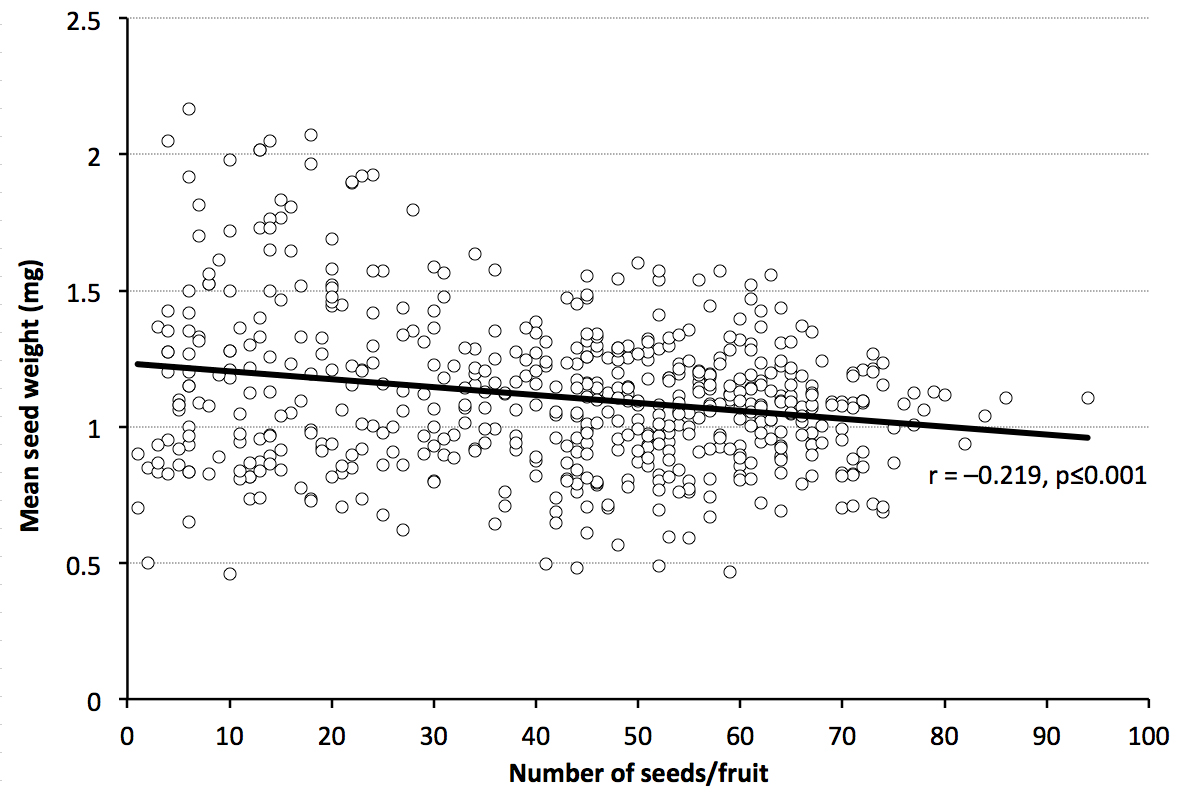


**Figure S5.** Pearson’s correlation (r = -0.22, *p*<0.001) between the number of seeds per fruit and the mean seed weight (mg) for all pollination treatments pooled.

**Table S6.** Within-population genetic variation of the natural F1 generation of the field population Gasteren. All reported values are means with their respective standard errors. Note that pin frequency is unknown, because the plants were juveniles and had not flowered yet.

| Population | N | Allelic richness | H_O_ | H_E_ | F_IS_ |
| --- | --- | --- | --- | --- | --- |
| Gasteren Field F1  SE | 10 | 1.67  0.19 | 0.22  0.08 | 0.25  0.07 | 0.14  0.14 |
